# Commencing open heart surgery in resource limited countries: lessons from the LASUTH experience

**DOI:** 10.11604/pamj.2014.19.105.4848

**Published:** 2014-09-29

**Authors:** Mobolaji Adewale Oludara, Jonathan Nwiloh, Adetokunbo Fabamwo, Phillip Adebola

**Affiliations:** 1 Department of Surgery, Lagos State University Teaching Hospital, Ikeja, Lagos, Nigeria; 2 Department of Surgery, Atlanta Medical Center, Atlanta, USA; 3 Directorate of Clinical Services, Lagos State University Teaching Hospital, Ikeja, Nigeria; 4 Department of Medicine, Lagos State University Teaching Hospital, Ikeja, Lagos, Nigeria

**Keywords:** Open heart surgery, developing countries, lessons

## Abstract

The challenge of commencing cardiac surgery in developing countries of Africa is onerous. We present a model from the experience of carrying out open cardiac surgical procedures at the Lagos State University Teaching Hospital (LASUTH) with three separate missions between 2004 and 2006. This paper details the challenges of starting open heart surgery in a resource limited environment. We propose that owing to the huge financial investment needed, government sponsorship as well as collaboration with overseas based and local non-governmental agencies may be required to jump start the process of open cardiac surgery. Local staff training opportunities are also provided by such missions and this can further be complemented by overseas exposure in areas of need for capacity building. In our centre, the initial investment has led to the recruitment of additional trained staff including 2 cardiothoracic surgeons. Further benefits of training of 2 perfusionists and a nurse has improved capacity in cardiac surgery service at our center.

## Introduction

World wide there is a huge burden of death from cardiovascular disease [1]. In more advanced environments surgical intervention by open heart procedures has significantly led to the reduction in the fatality of these conditions. Surgery for congenital and acquired cardiac diseases was first performed in LASUTH in December 2004. Prior to this, various ad hoc arrangements anchored by overseas based non governmental organisations and in partnership with government were the only alternatives in the search for surgical intervention where indicated in patients with congenital and acquired cardiac conditions. Many other patients in Lagos, and in Nigeria remained with their morbid conditions and had to make do with mere medical treatment of the various complications associated with their cardiac ailment including recurrent chest infections, heart failure and endocarditis [2, 3]. Earlier successful efforts at open heart surgery across the West African sub region were mainly at the University of Nigeria Teaching Hospital, Enugu [4], Korle Bu Teaching hospital in Accra, Ghana [3] as well as cases done in Abidjan, Ivory Coast. These efforts, laudable as they were remained largely incapable of addressing the disease burden across the sub region. At the Lagos State University Teaching hospital (LASUTH), an America based non governmental organization (Global Eagle foundation) in collaboration with the Lagos state government sponsored the commencement of open heart surgery in a medical mission in December 2004. During this exercise and subsequent ones in 2005 and 2006, various septal and valvular defects amongst other intra-cardiac problems were corrected with a total of twenty-four open heart procedures and two other thoracic cardiovascular procedures carried out. Two patients died. Subsequent impact of the commencement of this programme is the attraction of additional trained staff (2 Cardiothoracic Surgeons) and the training of other local staff (Perfusionists and Intensive Care and Cardiac operating room Nurses) and the maintenance of a Critical Care Unit-CCU) with the overall result that regular elective cardiac surgery is now taking place at LASUTH today. If the cardiac surgical needs of an environment are to be addressed, a good knowledge of the local terrain as well as anticipated geographical, financial and demographic limitations are required [5–7]. In an earlier report from other workers in a developing country, the critical importance of teamwork, collaboration between all groups involved and proper program organization were identified as contributory to overall success [8]. In this paper we present a profile of the practical steps taken to facilitate the take off of open heart surgery at LASUTH, Lagos, Nigeria.

### LASUTH experience and preparation guidelines

#### Background initiatives

The Lagos state government in partnership with Global Eagle Foundation- an American based non government organization sponsored three cardiac missions between 2004 and 2006. Some equipment including a bypass machine were donated by the Global Eagle Foundation. A Planning Committee was set up by the government. Members included top hospital management, anaesthetists, surgeons, cardiologists, nurses laboratory scientists, pharmacists as well as some Ministry of Health staff. The initial preparation included upgrading of the physical infrastructure like the operating theatre and the intensive care unit, assembly of all the required medical equipment, training of the local health personnel and recruitment of patients.

#### LASUTH outcome

Between 2004 and 2006 a total of 24 cases of open heart surgery were performed at LASUTH. The mean age of the cases performed was 28yrs with a range from 10 to 50yrs. There were 13 males and 11 females. Of the total of 24 cases, 12 had valve replacement surgery (aortic and mitral), 11 cases had septal defect repair (atrial septal and ventricular septal) and one case of left atrial myxoma excision. The average duration of hospital admission was 14 days with a range of 7 to 27 days. There were 2 mortalities recorded (one case of mitral valve replacement and another case of closure of ventricular septal defect VSD who was found at surgery to have a pre-operatively undetected patent ductus arteriosus PDA). Today, open cardiac surgery is routinely performed at LASUTH but the initial commencement in 2004 necessitated considerable changes and preparation

#### Hospital infrastructure

A decision was made to rebuild an open ward bungalow building sited within the hospital. The rebuilding process took place under the guidance of the planning committee over the period spanning July to November 2004. An operating room, an intensive care unit (ICU) with four beds and in patient amenity wards with four admission facilities were carved out of the bungalow. The structure was then connected to the main hospital electricity supply as well as a powerful electricity generator. Because of the nature of the proposed surgeries which would not give room for any interruption of power supply, the surgical lists were run always on the alternate power generating system. Piped gases were routed into both OR and ICU as well as the amenity wards. Other areas built into the facility included a side laboratory for urgent blood gas analyses as well as other haematological and biochemical tests. There was also a Nurses station, Pharmacist section, equipment store reception, staff lounge and office for the Coordinator of the ICU.

#### Equipment

The donation of equipment by the Eagle Foundation helped to encourage a good take off of the project. One bypass machine was donated out of the two that were brought. Other equipments donated included cardiac monitors, 2 ventilators, infusion pumps, pneumatic sternal saw, consumables for the bypass machine, consumables for cardiac surgery, including venous canullae aortic canullae, cardioplegia infusion circuit, synthetic patch materials (haemashield, Dacron, PTFE), metal valves for aortic and mitral valve surgery, catheters for chest drainage, suture materials, including steel wires for closure of sternotomy wound. A standard instrument tray for open heart surgery was provided for use with a spare tray.

#### The team

The team was drawn from the visiting American group from Global Eagle Foundation and ananaesthetist based in the United Kingdom (UK) and supported by the local based experts (2 General Surgeons with experience in cardiothoracic surgery and a Cardiologist). The details are as follows. 1 Visiting Surgeon, 2 local Surgical assistants (later 3), 1 Anaesthetist,1 Perfusionist, 2 Operating room (OR) nurses, 2 intensive care (ICU) nurses.

#### Support services

There was huge support provided from government through the Lagos state Ministry of Health and the hospital management. This included the domestic hospitality services, transport, security and general hospitality for the visiting team members. Professionally, registration with the necessary medical and nursing councils were completed by the hospital management. Quality and speed of service in the main hospital laboratories were augmented both in haematology and clinical chemistry. Performance of International normalized ratio (INR) was established and arrangement was made for the provision of fresh frozen plasma from another Lagos state hospital that heads the state haematology section. The Pharmacy department also procured stock of necessary drugs in anticipation of the type of procedures to be performed.

#### Case selection

There was a general agreement to take on cases with less morbidity especially those without signs of advanced cardiovascular problems. Simple adult cases that were relatively physically fit with minimal or absent comorbidities were considered. It was not possible to consider paediatric cases due to lack of paediatric cardiopulmonary bypass machine. Two levels of screening were conducted, the first one by the local cardiologist and the second by the visiting cardiothoracic surgeon. The clinical histories and physical examination findings were reviewed along with the echocardiogram and the electrocardiographic findings. Pulmonary artery pressure changes were observed, to exclude those patients with complicated Eissenmenger transformation in septal defect anomalies. Transvalvular changes were also observed to identify those that could benefit from valve replacement surgery. Other haematological, biochemical and radiological investigations were also carefully assessed to rule out comorbidities.

#### Local training

Apart from the assisting surgeon and intensivist who already had overseas exposure to cardiac surgery, the local team members required major training prior to the first mission in 2004 and in subsequent missions. Lectures were given on Preparation for Cardiac surgery to familiarise the team with what to expect. A group of 10 nurses were selected to be trained as ICU nurses. This training which was made up of one week didactic lectures at LASUTH and practical sessions at both LASUTH and the University College Hospital, Ibadan was locally organized and coordinated by the local team assisted by an experienced American trained ICU nurse. In the course of the subsequent missions they were then able to get more experience. From the time of the first mission, a graduate of Physics showed interest in perfusion work and worked closely with the visiting team perfusionist. He was later sent to cardiothoracic surgical unit in Scotland for further training.

#### Cost of service

The early effort at cardiac surgery in LASUTH were largely supported by the material donations given by the overseas NGO and the funds from the Lagos state government. The next phase was to evolve a costing system that would take into account the various consumables used in cardiac surgery finding a way of recovering the cost either by direct payment from patients or their sponsors or through health insurance schemes. This approach is currently being used to sustain the procedures by the new team that succeeded the Missions team

## Discussion

In the process of commencement of a new set of procedures in surgical practice, particularly when it involves high level surgery as in open heart operations or in minimal access surgery, two clearly distinct methods often come into the fore[5, 9]. The first is to support a trainee surgeon at advanced level of training to travel overseas for more training with the hope that on return he will set up the intended programme by locally training other doctors and paramedical staff. Open heart surgery is a team work involving the Cardiac surgeon, Cardiac Anaesthetist, Perfusionist, Cardiac operating room nurses, Intensive care nurse and the Doctor intensivist. This primary approach thus requires careful planning and monetary investments as well as time duration to achieve. The drawback or problems with this approach is that many young trainees sent overseas do often end up permanently re-locating there, thereby truncating the intention of setting up a programme locally. The other approach is to invite foreign experts to team up with the local personnel in ‘surgical missions’ during which the home team is gradually trained and expected to continue the programme after the period of missions. At LASUTH, commencement of open heart surgery was by a combination of the first and second methods. A few of the local personnel already had experience in open heart surgery at advanced centers in the United Kingdom, South Africa and the United States of America, thus facilitating the collaboration with the visiting American expert team.

In any open heart surgery programme, two major areas of concern remain the structure and functionality of the operating theatre and the intensive care units[1, 9, 10]. It was along this vision that a purpose designed refurbishment of one of the hospital wards led to the creation of the desired operating room and intensive care unit for the programme. The theatre space must be adequate enough not only to accommodate the surgical team and OR nurses, but also a team of anaesthetists as well as the Perfusionist and his assistants. The supporting equipment including heart lung bypass machine will be accommodated as well. Adequate efforts to identify a conducive theatre environment is a fundamental basic towards success. Investments in the purchase of instruments and the equipment for cardio-respiratory support in the ICU including ventilators, intra aortic balloon pumps counterpulsation as well as equipment for close monitoring, invasive and non invasive treatments remain key challenges in a programme of this nature. The LASUTH programme had a good take off through donation of these equipment by the NGO. Availability of drugs and consumables is another key area of preparation [11]. Potent and unexpired drugs including inotropes, antiarrhythmic drugs as well as cardioplegia solutions and consumables like venous and arterial canullae, prosthetic valves, synthetic grafts for septal defect closure must be of the highest quality. Owing to a good collaboration established with the hospital drug Quality Control Unit the potency of drugs donated by the NGO and those purchased by the government were duly validated.

High level training of personnel is obligatory to success in this kind of programme, as avoidable mistakes could lead to fatalities [12]. Being a new programme steps should be taken at every stage to minimise the tendency towards clinical errors, accidents or mistakes which could be unforgiving in their negative or adverse effects. The local experts were able to conduct rigorous training of 10 staff nurses for ICU care as well as delivering didactic lectures on what to expect and which roles were expected from the various professional groups before, during and after open heart surgery. Training of laboratory workers and provision of a side laboratory next to the operating theatre where blood gases can be done was a major component of the preparation. The LASUTH laboratory teamed up with the state haematology department domiciled at the General hospital, Marina, Lagos for experience in performing international normalized ratio (INR) on blood samples and also the provision of fresh frozen plasma (FFP) as well as other forms of laboratory support. This is a primary area of concern both during and after surgery when anticoagulants will be used and the patient require good monitoring and sometimes intervention with blood products. On the need for good case selection and pre-operative preparation, diagnosis was conducted using the available real time transthoracic echocardiogram. As a newly developing programme and in the absence of cardiac catheterization laboratory, pulmonary arterial pressure measurements may be inaccurate and such errors could lead to intra-operative problems where elevated pulmonary pressures could lead to major perioperative morbidity and mortalities [11]. This could be seen in patients developing an Eissenmenger complex in long standing cases of septal defects progressing to a reversed shunt flow. Pre-operative risk assessment using the New York heart disease classification helped to identify cases that stand good chances of successful surgical outcomes.

## Conclusion

Any open heart surgery programme is cost intensive. However using the model of collaboration between overseas NGO's and the Lagos state government, it is needful for low resource countries to seek such collaboration to lessen the financial burden when planning to start open heart surgery programmes. A major determinant of success in the LASUTH case is the priority given to the programme by the government. This translated to the government taking up the expenses and financial burden off the 24 patients operated upon between 2004 and 2006.
